# Developing the Clarity and Openness in Reporting: E3-based (CORE) Reference user manual for creation of clinical study reports in the era of clinical trial transparency

**DOI:** 10.1186/s41073-016-0009-4

**Published:** 2016-05-03

**Authors:** Samina Hamilton, Aaron B. Bernstein, Graham Blakey, Vivien Fagan, Tracy Farrow, Debbie Jordan, Walther Seiler, Anna Shannon, Art Gertel, for the Budapest Working Group

**Affiliations:** 1 European Medical Writers Association, Chester House, 68 Chestergate, Macclesfield, Cheshire SK11 6DY UK; 2 American Medical Writers Association, 30 W. Gude Drive, Ste. 525, Rockville, MD 20850-4347 USA; 3 Statisticians in the Pharmaceutical Industry, Durford Mill, Petersfield, Hampshire GU31 5AZ UK; 4 Sam Hamilton Medical Writing Services Limited, Newcastle Upon Tyne, UK; 5 Aaron Bernstein Consulting, LLC, Millburn, NJ USA; 6 Consult2Deliver, BioCity, Pennyfoot Street, Nottingham, NG1 1GF UK; 7 Quintiles, The Alba Campus, Rosebank, Livingston, West Lothian EH54 7EG UK; 8 PPD, Granta Park, Great Abingdon, Cambridge, CB21 6GQ UK; 9 Debbie Jordan Limited, Hook, Hampshire UK; 10 Bayer Pharma AG, 13342 Berlin, Germany; 11 Quintiles, 500 Brook Drive, Green Park, Reading, Berkshire RG2 6UU UK; 12 MedSciCom, LLC, Lebanon, NJ USA

**Keywords:** Reporting guidelines, Reporting standards, Randomised controlled trials, Information dissemination, Disclosure

## Abstract

**Background:**

Interventional clinical studies conducted in the regulated drug research environment are reported using International Council for Harmonisation (ICH) regulatory guidance documents: ICH E3 on the structure and content of clinical study reports (CSRs) published in 1995 and ICH E3 supplementary Questions & Answers (Q & A) published in 2012.

Since the ICH guidance documents were published, there has been heightened awareness of the importance of disclosure of clinical study results. The use of the CSR as a key source document to fulfil emerging obligations has resulted in a re-examination of how ICH guidelines are applied in CSR preparation. The dynamic regulatory and modern drug development environments create emerging reporting challenges.

**Methods:**

Regulatory medical writing and statistical professionals developed Clarity and Openness in Reporting: E3-based (CORE) Reference over a 2-year period. Stakeholders contributing expertise included a global industry association, regulatory agency, patient advocate, academic and Principal Investigator representatives.

**Results:**

CORE Reference should help authors navigate relevant guidelines as they create CSR content relevant for today’s studies. It offers practical suggestions for developing CSRs that will require minimum redaction and modification prior to public disclosure.

CORE Reference comprises a Preface, followed by the actual resource. The Preface clarifies intended use and underlying principles that inform resource utility. The Preface lists references contributing to development of the resource, which broadly fall into ‘regulatory’ and ‘public disclosure’ categories. The resource includes ICH E3 guidance text, ICH E3 Q & A 2012-derived guidance text and CORE Reference text, distinguished from one another through the use of shading. Rationale comments are used throughout for clarification purposes.

A separate mapping tool comparing ICH E3 sectional structure and CORE Reference sectional structure is also provided.

Together, CORE Reference and the mapping tool constitute the user manual.

**Conclusions:**

This publication is intended to enhance the use, understanding and dissemination of CORE Reference.

The CORE Reference user manual and the associated website (http://www.core-reference.org) should improve the reporting of interventional clinical studies.

Periodic updates of CORE Reference are planned to maintain its relevance.

**Registration:**

CORE Reference was registered with http://www.equator-network.org on 23 March 2015.

## Background

The current International Council for Harmonisation (ICH) regulatory guidance documents for clinical study reports (CSRs) are ICH E3 [1] (effective 1995) and the ICH E3 2012 supplementary Questions & Answers (Q & A) document [2]. The 2012 Q & A document addresses some of the ambiguity inherent to ICH E3. Interpretational texts on the effective use of clinical regulatory guidance documents form an integral part of the clarifying process for guideline end-users [3–5]. We similarly intend Clarity and Openness in Reporting: E3-based (CORE) Reference to provide interpretational support of the guidelines and pragmatic suggestions for preparing CSRs.

In addition, recently mandated public disclosure of CSRs submitted in Europe for regulatory review requires [6] that the clinical research community understands the complex concepts around public disclosure of clinical-regulatory documents and implements appropriate practical solutions in order to minimise associated risks, which are largely privacy-related [7]. The CSR is the first clinical-regulatory document type to be disclosed. The experiences of both applicants and regulators in these formative times are expected to shape future direction in this area. Therefore, resources that support this early learning process should be of value to the global clinical research community.

The objective of this project was to create a user manual to help medical writers navigate relevant guidelines as they create CSR content for today’s studies. The main resource, CORE Reference, was based on the long-standing experiences of clinical research professionals who report clinical studies using the ICH guidelines, and extensive literature review, followed by a structured approach to develop internationally based consensus. The findings were aggregated and include extensive information to support resource utility, detailed content suggestions and practical suggestions for developing CSRs that will require minimum redaction and modification prior to public disclosure. A separate mapping tool comparing ICH E3 and CORE Reference sectional structure supports the resource. CORE Reference and the mapping tool constitute the user manual.

## Methods

### Composition of the BWG

In May 2014, at the European Medical Writers Association (EMWA) Conference in Budapest, the lead author of CORE Reference and EMWA Vice President (SH) convened the Budapest Working Group (BWG), a group of experts from the Medical Writing community, to address current controversies and limitations in the field of reporting clinical studies and offer potential solutions. The BWG are the authors of CORE Reference and of this publication and include members of EMWA and the American Medical Writers Association (AMWA) to ensure representation for the EU and USA. The group comprises experts in ICH E3, CSR templates, CSR authoring and the public disclosure of clinical-regulatory documents. These individuals are employees of a pharmaceutical company, contract research organisations and freelancers, brought together in an attempt to represent the range of perspectives of professionals commonly engaged in authoring clinical-regulatory documents. A statistician and clinical pharmacologist, both members of Statisticians in the Pharmaceutical Industry (PSI), joined the BWG in June 2014, to ensure that all areas have expert input. There was no official involvement of PSI. In December 2014, the first open-access publication about this project was published [8]. In February 2015, AMWA officially joined EMWA as equal partners, and shortly afterwards, CORE Reference was registered with EQUATOR [9]. All authors gave their time and expertise to this project, voluntarily and without financial compensation, in the belief that an open-access user manual to support clinical study reporting would benefit today’s healthcare industry. Further information on individual author contributions is included in the ‘Authors’ information’ section.

### Project plan

In the original project plan [8], we described our intention to review the existing guidelines for CSR and clinical study protocol (CSP) authoring and develop internationally based consensus by involvement of relevant stakeholders. The planning and conduct of this project closely followed the AGREE II Instrument recommendations [10]. During the 2-year project, the BWG took a pragmatic and responsive approach to challenges presented by a dynamic professional environment.

Emerging awareness of a parallel and more mature CSP effort [11] prompted the decision to discontinue our planned CSP workstream and instead support the efforts of colleagues outside our group.

The 2-year Roadmap [8] summarises the BWG’s planned and conducted *de novo* and oversight reviews. The stakeholders (described in detail below) included representatives from a global industry association, regulatory agency, patient advocacy group, academic and Principal Investigator, who reviewed the CORE Reference resources, and provided insights and analysis over the period March 2015 to February 2016.

Multiple, extensive and rigorous literature searches were conducted throughout the 2-year project to support the broad aim of integrating relevant global and regional (EU and USA) regulatory guidance into the resource. Due diligence was exercised throughout and to the best ability of the BWG.

### Development of the user manual

The original work package submitted for stakeholder comment in March 2015 took the form of separate concurrently prepared ‘Text’ (i.e. recommendations for CSR authoring) and ‘Rationales’ (i.e. rationales for and clarifications of the reasons for each recommendation) documents, as well as a mapping tool. The mapping tool, prepared on completion of the Text and Rationales documents, compared ICH E3 and CORE Reference sectional numbering. The original Text document included a limited introduction section. During the stakeholder comment period (March to June 2015), the BWG decided that a single consolidated ‘Text and Rationales’ document prefaced by a scope-enhanced introduction would improve understanding and utility of the final resource. BWG progressed this reconstruction during the stakeholder comment period, during which the stakeholders independently arrived at a similar conclusion, and recommended to the BWG a merging of the planned Text and Rationales documents. The final published user manual comprises CORE Reference (a single document including an extensive ‘Preface’ followed by merged text, rationales and clarifications), as well as the separate mapping tool. Stakeholders took a further opportunity to comment on a pre-publication version of CORE Reference in February 2016. As a result, further format enhancements were made. Finally, in March 2016, following EMA’s publication of guidance [7] on the implementation of Policy 0070 [6], the BWG updated CORE Reference accordingly. The period March 2016 to the end of April 2016 was spent finalising (including quality control and proofreading steps) the user manual and website and this publication.

To allow rapid dissemination of periodic project updates, the planned December 2015 publication [8] was replaced with online video posting of project updates presented at the EMWA conferences in Dublin (06 May 2015) [12] and The Hague (06 November 2015) [13]. A similar presentation to that made in The Hague was made at the AMWA conference in San Antonio, TX, USA (01 October 2015).

The idea for a website to house the resource and support its utility, and logos to brand the resource and the BWG, came from an online search of similar projects. Many of the CORE Reference web design ideas were influenced by the website of the STROBE Statement [14].

Other than the described process and procedural changes to the original plans, the broad aims set out at the start of this project have been fulfilled through the final published resource, and the planned 2-year project timeline has been met. CORE Reference is available at http://www.core-reference.org. This publication describes the project and the launch of CORE Reference and is intended to enhance the use, understanding and dissemination of CORE Reference.

### Contributors to development of CORE Reference

#### Stakeholder: Health Canada

Consolidated comments for consideration were provided by Dr Celia Lourenco (Director, Bureau of Gastroenterology, Infection and Viral Diseases, Therapeutic Products Directorate Health Products and Food Branch), whose team of four contributors provided a high-level review of the documentation.

#### Stakeholder: Drug Information Association Medical Writing Community

The Community Chair, David Clemow (Lilly USA, LLC), on behalf of the Community’s 18-member CORE Review Task Force, provided anonymised comments from the task force members for consideration (several of whom had served on the ICH E3 2012 Q & A Implementation Working Group).

#### Stakeholder: Academic and Principal Investigator

Todd E. Pesavento, MD, Professor of Medicine, Medical Director of Solid Organ Transplantation, Ohio State University, USA, provided detailed comments for consideration.

#### Stakeholder: Patient advocate

David Gilbert, InHealth Associates, UK, provided high-level comments for consideration.

## Results

### Broad principles

**Fig. 1:**
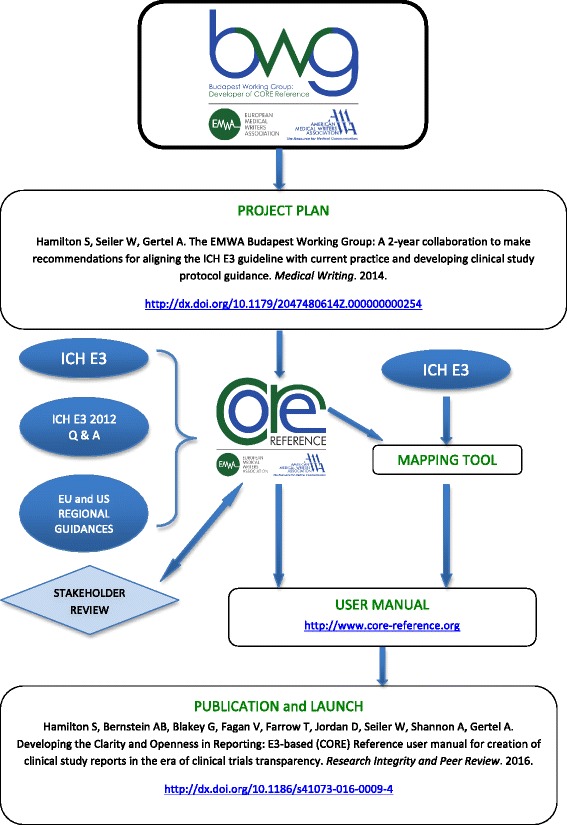
Process map of the CORE Reference project. *AMWA* American Medical Writers Association, *BWG* Budapest Working group, *CORE Reference* Clarity and Openness in Reporting: E3-based, *EMWA* European Medical Writers Association, *ICH* International Council for Harmonisation, *ICH E3* ICH Harmonised Tripartite Guideline: Structure and Content of Clinical Study Reports E3. Step 4. 30 Nov 1995; *ICH E3 2012 Q & A* ICH E3 Guideline: Structure and Content of Clinical Study Reports Questions & Answers (R1). 6 July 2012. 'Project Plan' publication: http://dx.doi.org/10.1179/2047480614Z.000000000254; 'Publication and Launch' publication: http://dx.doi.org/10.1186/s41073-016-0009-4

CORE Reference and the mapping tool constitute the user manual (Fig. 1). CORE Reference is provided as a PDF. The separate mapping tool comparing ICH E3 sectional structure and CORE Reference sectional structure is provided in spreadsheet format to support its utility.

CORE Reference presents content suggestions and best practices that add value for medical writers creating ICH-compliant CSRs, but these come with the caveat that they may not work in all situations. CSR authors should use their judgement and, above all, make sensible structuring choices based on their particular study. This means that CORE Reference is a user manual rather than a template. In addition, although we intend CORE Reference to be globally relevant, we include links to relevant regional (EU and USA) guidance and other useful resources where possible, with explanation, to maximise utility of the resource. Consultation with the relevant regulatory agency or body is highly recommended in cases where there is doubt.

The latest available guidance on public disclosure of clinical-regulatory documents [6, 7] are integrated into CORE Reference through discrete colour-coded comments prompting the user to consider both the ‘primary use’ (i.e. for regulatory review) and ‘secondary use’ (i.e. for public disclosure) CSR. These discrete comments incorporated into CORE Reference should help CSR authors make informed choices as they navigate the evolving and complex area of redaction of sensitive information prior to public disclosure.

### CORE Reference: Clarity

ICH E3 and ICH E3 2012 Q & A update guidance text is presented in CORE Reference. Commonly perceived ambiguities in the ICH E3 guidance that were not addressed by the ICH E3 2012 Q & A update are addressed in CORE Reference. In addition, relevant regional (EU and USA) regulatory guidance are included. Further value-added insights, based on the extensive collective experience of the BWG and stakeholders, are included.

CORE Reference comprises ICH E3 guidance text, ICH E3 Q & A 2012-derived guidance text and CORE Reference text, distinguished from one another through the use of monochrome shading. All ICH E3 guidance text is either included as original wording; or is included as modified wording and the modification is explained; or is omitted, the omission is shown and the reason for the omission is explained. All ICH E3 Q & A 2012-derived guidance text is included and explained. Rationale comments—in ‘comment balloon’ format on the right-hand side of each page—are used for explanation and clarification purposes. A key explaining text shading and comments is included in the footer of each page of CORE Reference.

Where alternative presentations of the same information would work equally well in a CSR, they are shown with an explanation provided in the Rationale comments to allow CSR authors to make informed authoring choices relevant for their particular study.

A separate mapping tool comparing ICH E3 sectional structure and CORE Reference sectional structure is also provided to support the utility of CORE Reference.

CORE Reference technical format supports on-screen and print readability, with even the most basic printer hardware.

### CORE Reference: Openness

The global pharmaceutical industry agrees that the principles of responsible clinical trial data sharing must be upheld. The Pharmaceutical Research and Manufacturers of America (PhRMA) and the European Federation of Pharmaceutical Industries and Associations (EFPIA) issued joint Principles for Responsible Clinical Trial Data Sharing in January 2014 [15], followed by the Institute of Medicine who issued their report ‘Sharing Clinical Trial Data: Maximizing Benefits, Minimizing Risk’ in January 2015 [16]. The World Health Organization (WHO) encourages data-sharing initiatives in their April 2015 statement ‘Public Disclosure of Clinical Trial Results’ [17]. Further, in January 2016, the International Committee of Medical Journal Editors proposed manuscript publication requirements to help meet the obligation to responsibly share data generated by interventional clinical trials [18].

Initial limited public disclosure of study data is ongoing, with potential for expansion, in some parts of the world. The European Commission Guidance of 2006 requires mandatory posting of clinical trial results using the European Union Drug Regulating Authorities Clinical Trials database [19]. Title VIII of the Food and Drug Administration Amendments Act of 2007 [20] requires the registration and submission of summary results information to *ClinicalTrials.gov* for certain clinical trials of drugs (including biologic products) and devices. Changes to the rule to ‘…enhance patient enrolment, provide a mechanism to track subsequent progress of clinical trials, provide more complete results information, and enhance patient access to and understanding of the results of clinical trials’ were proposed on 21 November 2014 and opened for public comment (period ended 23 March 2015) [21]. There is no published timetable for enactment of changes to the rule.

With respect to privacy protections, selected references are presented below. In the USA, the Health Insurance Portability and Accountability Act (HIPAA) governs protected health information [22, 23], and the Department of Health and Human Services has also issued guidance [24]. In the European Union, Directive 95/46/EC of the European Parliament and of the Council covers protection of personal information [25]. In addition, the World Medical Association has issued guidance on privacy [26], and the Council of Europe Convention for the Protection of Individuals with regard to Automatic Processing of Personal Data also addresses protections [27]. The International Organization for Standardization (ISO)/TS 14265:2011 [28] provides a framework for classifying the various specific purposes that can be defined and used by individual policy domains (e.g. healthcare organisations, regional health authorities, jurisdiction countries) as an aid to the consistent management of information in the delivery of healthcare services and for the communication of electronic health records across organisational and jurisdictional boundaries. In Canada, the Health Privacy Act 2011–2012 [29] governs disclosure of personal information.

There are many country- and region-specific legislative directives that address protection of personal information. Some elements may not be entirely consistent; however, the general governing principles are very similar. There are other privacy protections that apply according to local jurisdiction, and we strongly advise to review the guidance(s) in force in applicable regions.

Requirements for broader public disclosure of trial data are mandated through the European Medicines Agency (EMA) Policy 0070 on publication of clinical data for medicinal products for human use [6] since 01 January 2015. Policy 0070 mandates that from 01 July 2015, CSRs from extension of indication and line extension applications are made publicly available, bringing them into alignment with CSRs in new marketing authorisation applications, which were made publicly available from 01 January 2015.

Sponsors may take differing approaches to ensure their CSRs comply with evolving responsible data sharing requirements and industry standards. Using CORE Reference as a resource for CSR authoring is one way to encourage sharing of best practices.

Increased requirements for data disclosure through CSR publication mean that medical writers must now consider the CSR as a single document with two uses, each with a distinct purpose and audience:

- The ‘primary use CSR’ (the EMA term for which is scientific review version^1^) is a technical document for regulatory review and comprises full CSR text and all CSR appendices. The information reported must not constrain the review process.
- The ‘secondary use CSR’ (the EMA term for which is redacted clinical report^1^) is for public disclosure and comprises redacted CSR text and selected appendices. Sensitive information presented in the ‘primary use CSR’ is redacted in the ‘secondary use CSR’.

The technical ‘primary use CSR’ has a primary audience comprising regulators and pharmaceutical industry professionals, and its derived and summary information are of interest to health technology assessment and marketing professionals. CSRs must support the decision-making process for the licensing of medicines, and the data reported must not constrain this process. The CSR also has a secondary audience associated with public disclosure following the marketing authorisation decision. Some data germane to regulatory review—and presented in the ‘primary use CSR’—may present risks if released directly into the public domain. This creates a need for a ‘secondary use CSR’ in which sensitive data are redacted, as described in the EMA’s March 2016 guidance on implementation of Policy 0070 [7]. The risks are largely associated with privacy. In addition, any single CSR represents only a portion of the data accrued in the course of research and development and may not represent the aggregate knowledge associated with the new medicine or indication.

If, in the ‘primary use CSR’, subject data are reported with care to protect sensitive information, this will streamline redaction in the ‘secondary use CSR’. This ‘proactive’ approach, first proposed by the BWG [8], is encouraged by the March 2016 EMA guidance on implementation of Policy 0070 [7]. These principles underlie CORE Reference recommendations and suggestions. CORE Reference guides the CSR author on creation of the ‘primary use CSR’ text, including practical suggestions for safeguarding patient anonymity and also on protection of the identity of individuals involved in the management, conduct and reporting of clinical studies, in such a way as to not constrain the process of regulatory review of the ‘primary use CSR’. CORE Reference gives separate attention to the topic of redaction of sensitive data necessary for creation of a ‘secondary use CSR’ fit for public audiences. Where it is possible to annex or append data in the ‘primary use CSR’ that may require subsequent redaction, practical placement suggestions are made to minimise or avoid altogether the piecemeal redaction of data in the CSR body that might otherwise be necessary.

### CORE Reference: Reporting: E3-based

The ICH E3 Q & A 2012 [2] document restates unequivocally that ICH E3 [1] is a guidance document and not a template. Similarly, CORE Reference is a user manual and not a template. CORE Reference offers suggestions for content but does not mandate a particular sequence or organisation of the individual CSR sections. However, to allow easy mapping to the original ICH E3 guidance document and to avoid conflict with guidance documents that refer to ICH E3 sectional numbering, CORE Reference maintains the level 1 heading hierarchy of ICH E3. It remains at the author’s discretion to decide on the most appropriate CSR structure. The content suggestions are intended to facilitate optimal reporting for the extended range of study designs commonly encountered in modern drug development—in addition to the universal study design elements of safety and efficacy, as laid out in ICH E3.

A separate CORE Reference/ICH E3 sectional mapping tool is provided to help medical writers understand suggested placement of content.

## Discussion

In December 2014, 7 months into this 2-year project, we published our project plan [8], which included an explanation of our ‘proactive approach’ to the complex area of CSR disclosure: ‘Industry is currently debating a two-step process for submitting and then publishing clinical study results. The two-step process involves producing a submission-ready CSR that may contain data that must be removed after submission to produce the final disclosure-ready CSR. We propose that the CSR should be as disclosure-ready as possible from the outset to safeguard against inadvertent identification of participants, assure optimally timed public disclosure of clinical trial results, and be as cost efficient as possible.’ This ‘proactive approach’ aligns with recent EMA guidance [7] which states: ‘EMA understands that in an initial phase redaction techniques are likely to be used by applicants/marketing authorisation holders (MAHs), taking into account that for a certain period, pharmaceutical companies will have to anonymise their data retrospectively (reactive data anonymisation), i.e. after the clinical report has already been submitted for scientific review. Importantly, redaction alone is more likely to decrease the clinical utility of the data compared to other techniques. Therefore, EMA is of the view that applicants/MAHs, after experience has been accumulated in the de-identification of clinical reports, should transition to other anonymisation techniques that are more favoured in order to optimise the clinical usefulness of the data published (proactive data anonymisation). Pharmaceutical companies are encouraged to use these anonymisation techniques as soon as possible, whilst ensuring data anonymisation is achieved.’

CORE Reference should therefore not only expedite the move towards EMA’s intended goal of a heavily ‘proactive approach’ to anonymisation but may also contribute to increased trust derived from this approach, which should reduce the need for piecemeal redaction of individual words and phrases throughout a document.

In addition, time, money and cost savings in the development of CSRs for their primary and secondary uses should be possible [8].

Furthermore, CORE Reference should increase the quality of final CSRs and enhance consistency within and between sponsors. It may also benefit systematic reviewers in their use of CSRs, which will also contribute to the development of a trust-enhanced environment.

The website is fitted with separate download counters for CORE Reference and the mapping tool. Although this will enable us to monitor resource downloads, it is less easy to monitor the use of CORE Reference in practice. We therefore encourage user feedback via both a ‘Contact’ and a ‘Support’ page.

CORE Reference is open for comments for a 6-week period from its publication date. Comments may be submitted via http://www.core-reference.org.

To maintain its relevance, surveillance of the evolving regulatory and public disclosure landscapes will support periodic update of CORE Reference.

## Conclusions

This publication is intended to enhance the use, understanding and dissemination of CORE Reference.

The CORE Reference user manual and the associated website (http://www.core-reference.org) should improve the reporting of interventional clinical studies.

Periodic updates of CORE Reference are planned to maintain its relevance.

### Ethics approval and consent to participate

Not applicable.

### Consent for publication

Not applicable.

### Availability of data and materials

Not applicable.

## Abbreviations

AMWA: American Medical Writers Association
BWG: Budapest Working Group
CORE Reference: Clarity and Openness in Reporting: E3-based Reference
CSP: clinical study protocol
CSR: clinical study report
EFPIA: European Federation of Pharmaceutical Industries and Associations
EMA: European Medicines Agency
EMWA: European Medical Writers Association
HIPAA: Health Insurance Portability and Accountability Act of 1996
ICH: International Council for Harmonisation
ISO: International Organization for Standardization
MAH: marketing authorisation holder
PhRMA: Pharmaceutical Research and Manufacturers of America
PSI: Statisticians in the Pharmaceutical Industry
Q & A: questions and answers
WHO: World Health Organization

## Footnotes

1 External guidance on the implementation of the European Medicines Agency policy on the publication of clinical data for medicinal products for human use. 2 March 2016. (http://www.ema.europa.eu/docs/en_GB/document_library/Regulatory_and_procedural_guideline/2016/03/WC500202621.pdf [Accessed 04 March 2016]). Chapter 2, Section 3.3.1.9 states the cover letter including declaration will include ‘Confirmation that the clinical reports submitted for scientific evaluation are the same as that submitted for publication…except for the redactions’.
